# Community and health system factors associated with antiretroviral therapy initiation among men and women in Malawi: a mixed methods study exploring gender-specific barriers to care

**DOI:** 10.1093/inthealth/ihaa041

**Published:** 2020-08-25

**Authors:** Khumbo Phiri, Kaitlyn McBride, Corrina Moucheraud, Misheck Mphande, Kelvin Balakasi, Eric Lungu, Pericles Kalande, Risa M Hoffman, Kathryn Dovel

**Affiliations:** Partners in Hope, P.O. Box 302, Lilongwe, Malawi; Department of Health Policy and Management, UCLA Fielding School of Public Health, Los Angeles, USA; Department of Health Policy and Management, UCLA Fielding School of Public Health, Los Angeles, USA; Partners in Hope, P.O. Box 302, Lilongwe, Malawi; Partners in Hope, P.O. Box 302, Lilongwe, Malawi; Partners in Hope, P.O. Box 302, Lilongwe, Malawi; Partners in Hope, P.O. Box 302, Lilongwe, Malawi; Department of Medicine and Division of Infectious Diseases, David Geffen School of Medicine at University of California Los Angeles, Los Angeles, USA; Partners in Hope, P.O. Box 302, Lilongwe, Malawi; Department of Medicine and Division of Infectious Diseases, David Geffen School of Medicine at University of California Los Angeles, Los Angeles, USA

**Keywords:** ART initiation, community barriers, gender, health system barriers, sub-Saharan Africa

## Abstract

**Background:**

Although community and health system factors are known to be critical to timely antiretroviral therapy (ART) initiation, little is known about how they affect men and women.

**Methods:**

We examined community- and health system-level factors associated with ART initiation in Malawi and whether associations differ by gender; 312 ART initiates and 108 non-initiates completed a survey; a subset of 30 individuals completed an indepth interview. Quantitative data were analyzed using univariate and multivariate logistic regressions, with separate models by gender. Qualitative data were analyzed through constant comparison methods.

**Results:**

Among women, no community-level characteristics were associated with ART initiation in multivariable models; among men, receiving social support for HIV services (adjusted OR [AOR]=4.61; p<0.05) was associated with ART initiation. Two health system factors were associated with ART initiation among men and one for women: trust that accessing ART services would not lead to unwanted disclosure (women: AOR=4.51, p<0.01; men: AOR=1.71, p<0.01) and trust that clients were not turned away from ART services (men: 12.36, p=0.001).

**Conclusions:**

Qualitative data indicate that men were concerned about unwanted disclosure due to engaging in ART services and long waiting times for services. Interventions to remove health system barriers to ART services should be explored to promote social support among men.

## Introduction

Timely antiretroviral therapy (ART) initiation is critical to curbing the HIV pandemic. Currently, WHO guidelines for adults and adolescents recommend initiating ART immediately (or as soon as possible) after HIV diagnosis to increase the uptake of ART and decrease the time to viral suppression for individual patients.^1^ Many countries in sub-Saharan Africa have adopted universal treatment policies,^2^ whereby ART is offered immediately following an HIV-positive test, regardless of CD4.^3^ Despite improvements in ART initiation under universal treatment policies,^4^^–^^7^ there are still significant gaps from diagnosis to initiation. In sub-Saharan Africa, studies have shown that up to 20–30% of identified HIV-positive individuals do not start treatment or encounter delays in treatment initiation.^8^^–^^10^ In Malawi, approximately 13% of individuals who test HIV-positive fail to initiate treatment,^11^ even though Malawi adopted a universal treatment policy in July 2016 and prioritizes same-day ART initiation.^12^

A number of studies in the sub-Saharan Africa region highlight the importance of community- and health system-level barriers to ART uptake,^13^^–^^15^ including fear of stigma and unwanted disclosure, long travel and wait times, as well as poor patient–provider interactions.^16^^,^^17^ However, evidence is lacking about how these factors influence ART initiation in the context of universal treatment and whether they differ from factors identified under guidelines that used CD4-guided or clinical criteria for ART initiation.

There are also limited data about if gender differences in community- and health system-level factors may be associated with ART initiation. For example, at the community-level, previous studies suggest that narrow and harmful gender norms may prevent men from initiating ART.^18^^–^^20^ On the other hand, fear of unwanted disclosure and stigma may be more pronounced for women who rely on spouses or sexual partners for economic support.^21^ At the health system-level, limited entry points for men, lack of male-friendly HIV services, long wait times and rigid clinic hours that clash with competing work demands may affect men's ART initiation,^22^ while lack of privacy within ART services and poor patient–provider interactions may be more impactful for women.

We use a mixed methods design to examine community and health system factors associated with ART initiation among men and women in Malawi under universal treatment policies. We examine correlates of initiation using quantitative data from recently diagnosed HIV-positive clients who initiated vs those who did not initiate ART, and qualitative interviews with a subset of these participants to explore how community and health system factors are perceived as influencing ART initiation. All analyses are disaggregated by gender.

## Methods

### Setting

Ten health centers and rural hospitals in central and southern Malawi were selected based on priority Presidential Emergency Plan For AIDS Relief (PEPFAR) sites^23^ that had a high HIV burden. Large referral hospitals were excluded from the study because they are not representative of most facilities in Malawi, and people who test positive at a referral hospital may opt to initiate ART at smaller, more convenient facilities. All selected facilities were supported by Partners in Hope, a non-profit organization that provides quality improvement strategies for HIV services through PEPFAR funding. Facilities varied by type/level and region (see Suppl A).

### Theoretical framework

We used the socioecological model (SEM) to explore community- and health system-level barriers to ART initiation for both men and women. The SEM provides an ideal framework for understanding how barriers across multiple levels of society may influence the utilization of care and health outcomes.^24^^–^^26^ The SEM framework identifies four levels of influence: (1) individual, (2) interpersonal, (3) community and (4) institutional (for our purposes, health systems).^27^ The SEM has been used previously in studies investigating factors influencing HIV-related outcomes, including ART initiation and adherence.^28^^–^^30^

### Design

We conducted a case control study: controls were defined as newly diagnosed HIV-positive clients who initiated ART within 14 days of receiving the diagnosis and returned for their 1 month follow-up ART appointment (hereafter referred to as ‘initiates’). Cases were defined as newly diagnosed HIV-positive clients who did not initiate ART within 14 days of receiving an HIV-positive diagnosis, or initiated ART but did not return for their 1 month follow-up ART appointment (hereafter referred to as ‘non-initiates’). We invited a subset of survey participants to participate in indepth interviews; we aimed for an equal number of initiates and non-initiates in these interviews balanced by gender. All data were collected between September 2016–June 2017.

### Participant recruitment

Initiates were recruited during standard ART clinic days through medical chart reviews completed by research assistants. Inclusion criteria included: (1) diagnosed as HIV-positive; (2) diagnosed after 1 July 2016, when the universal treatment policy was rolled out in Malawi; and (3) aged ≥18 years. Eligibility criteria were determined using a medical record review. Eligible individuals were approached to complete screening, written informed consent and data collection procedures. Any individual who was ineligible or refused to participate was replaced by the next ART client whose medical chart review indicated that they met eligibility criteria. All study activities for initiates took place in private rooms within the health facility.

Non-initiates were traced in the community by facility staff who provided routine tracing activities to encourage ART initiation (i.e. telephone calls and home visits). During these routine tracing activities, non-initiates were asked by facility staff if they were interested in participating in the study. Those who agreed were referred to a research assistant who completed the screening, written informed consent and data collection procedures, either that same day or on another day which was convenient for respondents. Any individual who was not traced, was ineligible or refused to participate was replaced by the next traced individual whose medical chart review indicated that they met the eligibility criteria. Nearly all study activities for non-initiates took place in respondents’ homes or at a private, convenient location chosen by the respondent.

### Data collection and analysis

#### Quantitative

A survey tool was developed using the SEM framework and existing literature. The tool asked questions about experiences in community- and health system-level variables and perceived barriers at each level. The survey tool included the following domains: demographic data, health facility characteristics, gender norms, as well as perceived barriers and facilitators to ART initiation. No identifiers were collected. Each survey lasted approximately 60 min.

For community-level influences on ART initiation, we included the following variables in our analysis: (1) talked frequently with someone currently on ART (vs not knowing anyone on ART, or knowing someone on ART but talking with them never/only once/occasionally); (2) disclosed their HIV-positive status to someone they talk with frequently (vs not disclosing their HIV-positive status to anyone, or disclosing it to someone but talking with them never/only once/occasionally); (3) received social support or encouragement from someone outside the health facility regarding accessing health services; and (4) gender norms, using the gender equitable men (GEM) scale used frequently throughout sub-Saharan Africa.^31^ The GEM has 18 questions regarding gender norms, with 5-point scale responses (ranging from strongly disagree to strongly agree); we used these to create an additive scale with scores ranging from 18 (highly gender equitable) to 90 (highly unequitable).

At the health system-level, we included four variables in our analysis: privacy (the respondent believes that their HIV status will not be disclosed at the health facility), stocks of medication (ART is always available at the health facility), consistent access to care (ART clients are not turned away from the health facility due to lack of provider availability) and travel time to the health facility (measured continuously [in min]). The first three health system variables were measured with 5-point scale responses (ranging from strongly disagree to strongly agree), which we collapsed into dichotomous variables that took a value of 1 for ‘strongly agree’ and 0 for all other response categories. We conducted sensitivity analyses with models using a broader operationalization, where 1 represented both ‘agree’ and ‘strongly agree’ and 0 represented ‘disagree’ and ‘strongly disagree’.

For all quantitative analyses, we included as covariates four individual-level variables: age (continuous), education (years of completed schooling, continuous), whether the respondent had worked for pay in the past month (yes/no) and a wealth index by taking the first component of a principal component analysis for a household asset index of 11 items^32^ (modified for the Malawi setting) and stratifying by quintiles. We included two interpersonal variables: the number of living children (continuous) and whether the respondent was sexually active (i.e. had a sexual partner in the last month, yes/no).

We conducted univariate logistic regression analyses to estimate the association between each SEM variable and initiation status (initiates vs non-initiates) stratified by gender. We conducted multiple logistic regressions, including factors within each level of the SEM framework (i.e. individual, interpersonal, community and health system). All models include fixed effects and clustered standard errors to account for sampling at the health facility level. All analyses were performed in Stata 14.2 (StataCorp College Station, TX, USA).

#### Qualitative

The indepth interview guide was developed using the SEM framework^33^ and existing literature.^34^^,^^35^ The guide included three domains: (1) sociodemographic data, (2) respondents’ lived experiences and the social context surrounding respondents’ decisions to initiate ART or not and (3) the specific barriers and facilitators to ART initiation experienced by respondents at each level of the SEM framework. Interviews were conducted by trained research assistants in the local language (Chichewa), were audio-recorded and lasted approximately 60 min each.

Interview data were transcribed verbatim and translated to English. Transcripts were analyzed in Atlas.ti 7.5^36^ using constant comparison methods.^37^ Deductive codes were developed based on the SEM framework and existing literature, and inductive codes were added as they emerged from the dataset. A codebook was developed and finalized after the first five transcripts were coded. Independent coding was completed by three authors (KP, MM and PK). Coding was reviewed by KD and any disagreements were resolved. For this analysis, we focus on codes within the community and health system levels of the SEM. We present dominant themes separately both for men and women and for initiates and non-initiates.

## Results

### Quantitative results

A total of 420 HIV-positive adults completed the survey, of whom 312 were initiates and 108 were non-initiates. Of the sample, 50% (n=212) were women and 19% (n=40) of those women were non-initiates. Men comprised the other 50% (n=212) of the total sample and 32% of those men (n=68) were non-initiates. Table 1 presents characteristics of initiates and non-initiates by gender.

**Table 1.** Characteristics of the initiates and non-initiates by level in the socioecological model, stratified by gender (n=420)

	Females		Males	
	Non-initiates	Initiates	Non-initiates	Initiates
	(n=40)	(n=172)	(n=68)	(n=140)
Individual				
Age (y), mean (SD)	33.6 (8.8)	37.2^‡^ (11.6)	37.3 (11.0)	39.9 (11.0)
Education (y), mean (SD)	3.8 (3.6)	3.7 (3.6)	4.8 (3.5)	5.1 (3.6)
Asset index quintile, mean (SD)	2.6 (1.4)	2.7 (1.4)	3.0 (1.5)	2.8 (1.4)
Currently/recently working, n (%)	12 (30.0%)	46 (26.7%)	19 (27.9%)	28 (20.0%)
Interpersonal				
Number of living children, mean (SD)	3.4 (1.9)	3.2 (2.2)	3.4 (2.4)	3.6 (2.3)
Sexually active, n (%)	31 (77.5%)	114 (66.3%)^‡^	62 (91.2%)	113 (80.7%)^‡^
Community				
Talk frequently with someone on ART, n (%)	19 (47.5%)	96 (55.8%)	26 (38.2%)	67 (47.9%)
Disclosed to someone they talk with frequently, n (%)	15 (37.5%)	96 (55.8%)^*^	21 (30.9%)	45 (32.1%)
Social support for seeking care, n (%)	27 (67.5%)	139 (80.8%)^*^	30 (44.1%)	88 (62.9%)^*^
Gender norms (GEM) score^b^, mean (SD)	57.0 (6.5)	55.8 (6.9)	49.3 (8.3)	47.3‡ (7.4)
Health Systems				
Privacy (HIV status will not be disclosed at health facility), n (%)	16 (40.0%)	102 (59.3%)^*^	40 (58.8%)	102 (72.9%)^*^
Medication stocks (ART are always available), n (%)	20 (50.0%)	110 (64.0%)	44 (64.7%)	128 (91.4%)^***^
Access to care (patients are not turned away), n (%)	19 (47.5%)	105 (61.1%)	37 (54.4%)	125 (89.3%)^***^
Travel time to health facility (min), mean (SD)	77.4 (73.1)	69.8 (59.2)	65.4 (65.9)	70.4 (56.0)

^‡^p<0.1; ^*^p<0.05; ^**^p<0.01; ^***^p<0.001.

p-value was calculated using t tests or χ^2^ tests for the comparison between female initiates and non-initiates or between male initiates and non-initiates score.

^a^Asset group developed based on principal component analysis incorporating variables about household ownership of metal roof, electricity, Koloboyi, paraffin lamp, radio, television, mobile phone, bed with mattress, sofa, table and chairs, and refrigerator.

^b^GEM score based on participant responses to questions about woman's role in the home, sexual relationships, interpersonal violence, household decision-making, masculine identity and male friendships.

At the community level, female initiates were more likely than female non-initiates to have disclosed their HIV status to someone they talked with frequently (55.8% vs 37.5%; p=0.04). Female initiates were more likely than female non-initiates to have received support from their social network regarding seeking health services, but this difference was not significant (80.8% vs 67.5%; p=0.07). At the health system level, female initiates were more likely than female non-initiates to believe that the health facility offered private ART services (59.3% vs 40.0%; p=0.03), with a non-significant trend towards female initiates to have more confidence in stocks of medications (64.0% vs 50.0%; p=0.10) and consistent access to HIV care (ART clients are not turned away; 61.1% vs 47.5%; p=0.12).

Male initiates were more likely than male non-initiates to have received support from their social network for seeking health services (62.9% vs 44.1%; p=0.01) and were more likely to believe the health facility offered private ART services (72.9% vs 58.8%; p=0.04), adequate stocks of medication (91.4% vs 64.7%; p<0.001) and consistent access to HIV care (89.3% vs 54.4%; p<0.001).

Table 2 presents modeled estimates of the community- and health system-level factors associated with ART initiation among men and women, controlling for individual- and interpersonal-level factors (age, education, household wealth, number of living children, employment status and whether the respondent was sexually active). For women, no community-level factors were significantly associated with ART initiation. At the health system level, female initiates were more likely than female non-initiates to believe the facility offered private ART services (adjusted OR [AOR]: 4.51; 95% CI 1.69 to 12.02).

**Table 2.** Community and health facility-level factors associated with initiating ART, stratified by gender and controlling for individual and interpersonal factors

	Females	Males
	AOR (95% CI) (n=212)	AOR (95% CI) (n=208)
Community		
Talk frequently with someone on ART, n (%)	1.41 (0.57 to 3.53)	1.39 (0.80 to 2.43)
Disclosed to someone they talk with frequently, n (%)	2.03 (0.66 to 6.22)	1.06 (0.39 to 2.86)
Social support for seeking care, n (%)	2.00 (0.64 to 6.28)	4.61^*^ (1.36 to 15.60)
Gender norms (GEM) score^a^, mean (IQR)	0.98 (0.92 to 1.05)	0.97 (0.94 to 1.01)
Health System		
Privacy (HIV status will not be disclosed at health facility), n (%)	4.51^**^ (1.69 to 12.02)	1.71^**^ (1.21 to 2.42)
Medication stock (ART are always available), n (%)	1.92^‡^ (0.90 to 4.08)	5.48^**^ (1.88 to 16.01)
Access (patients are not turned away), n (%)	2.57^‡^ (1.00 to 6.69)	12.36^***^ (6.46 to 23.66)
Travel time to health facility (min), mean (IQR)	1.00 (0.99 to 1.01)	1.00 (1.00 to 1.01)

^‡^p<0.1; ^*^p<0.05; ^**^p<0.01; ^***^p<0.001.

AOR's control for age (continuous), education (continuous), asset index (quintiles based on principal component analysis reflecting household ownership of metal roof, electricity, Koloboyi, paraffin lamp, radio, television, mobile phone, bed with mattress, sofa, table and chairs, and refrigerator), currently working, number of living children, sexually active and facility fixed effects.

^a^GEM score based on participant responses to questions about woman's role in the home, sexual relationships, interpersonal violence, household decision-making, masculine identity and male friendships.

Male ART initiates were more likely than male non-initiates to receive social support regarding accessing health services (AOR: 4.61; 95% CI 1.36 to 15.60) when controlling for individual- and interpersonal-level factors. All health system variables except distance to the health facility were positively associated with ART initiation among men. Male initiates were more likely than non-initiates to believe that the health facility offered private ART services (AOR: 1.71; 95% CI 1.21 to 2.42), adequate stocks of medication (AOR: 5.48; 95% CI 1.88 to 16.01) and consistent access to HIV care (AOR: 12.36; 95% CI 6.46 to 23.66).

Table 3 presents perceived barriers to ART services stratified by ART initiation and gender. Few women reported experiencing community- or health system-level barriers to ART initiation. Fear of disclosure to one's sexual partner was significantly lower among female initiates compared with female non-initiates (1.2% vs 7.5%; p=0.02) and perceived lack of privacy for ART services was significantly lower among female initiates than non-initiates (0% vs 7.5%; p<0.001).

**Table 3.** Reported barriers to facility-based ART services, stratified by gender

	Females		Males	
	Non-initiates	Initiates	Non-initiates	Initiates
	(n=40)	(n=172)	(n=68)	(n=140)
Community				
Fear of unwanted disclosure to sexual partner	3 (7.5%)	2 (1.2%)^*^	19 (27.9%)	14 (10.0%)^**^
Fear of unwanted disclosure to others (not sexual partner)	1 (2.5%)	3 (1.7%)	25 (36.8%)	39 (27.9%)
Travel due to work or social responsibilities (e.g. work, visiting family, traveling to a funeral)	4 (10.0%)	10 (5.8%)	20 (29.4%)	21 (15.0%)^*^
Health System				
Time required for ART visits	0 (0%)	0 (0%)	10 (14.7%)	6 (4.3%)^**^
Rude or mean healthcare workers	1 (2.5%)	2 (1.2%)	3 (4.4%)	2 (1.4%)
Lack of privacy at the health facility	3 (7.5%)	0 (0%)^***^	13 (19.1%)	20 (14.3%)
Distance to the health facility	7 (17.5%)	17 (9.9%)	15 (22.1%)	46 (32.9%)

^‡^p<0.1; ^*^p<0.05; ^**^p<0.01; ^***^p<0.001.

p-value calculated using t-tests or χ^2^ tests for the comparison between female initiates and non-initiates or between male initiates and non-initiates.

A larger proportion of men than women listed community- and health system-level barriers to ART initiation, in particular a fear of disclosure of their HIV status to people in the community (30.8%) and travel difficulties due to work or social responsibilities (19.7%). Compared with male non-initiates, male initiates were significantly less likely to report fear of disclosure to one's sexual partner (10.0% vs 27.9%; p=0.001) or travel as a barrier to care (15.0% vs 29.4%; p=0.01). At the health system level, male initiates were significantly less likely to mention not having time to visit health facilities compared with male non-initiates (4.3% vs 14.7%; p=0.01).

### Qualitative results

Thirty indepth interviews were completed. Eight interviews were excluded from analyses due to audio-recordings which were difficult to hear or interviews which were incomplete or interrupted. There were 14 interviews with initiates (14/22) and 10 interviews with non-initiates (10/22). Initiates were predominately female (8/14), had disclosed their HIV status to their partner (9/14) and felt healthy at the time of diagnosis (6/14). Non-initiates were predominately male (8/10), felt healthy at the time of diagnosis (10/10) and had disclosed their HIV status to their partner (5/10).

#### Community-level factors

Most men mentioned fear of unwanted disclosure due to anticipated stigma, while this issue was mentioned by only a handful of women. Some men who did not initiate ART discussed their concerns related to stigma and how they felt shame in revealing their diagnosis.

*It is not good [to disclose], usually. This is a shameful disease so disclosing to others is not a good idea* (non-initiate, 38-years-old male).

*I thought it would not be good to start publicizing considering the life of people here in the village… A lot of people have prejudice* (non-initiate, 26-years-old male).

For both men and women, those who were concerned about anticipated stigma from community members were usually most concerned about gossip and slandering from friends and acquaintances due to their HIV-positive status.

*I never [disclosed]… because this is a very confidential matter. So once you tell other people especially those who are not psychologically mature, they [community members] will start saying a lot of things which will cause psychological pressure to you and as such your life is affected* (non-initiate, 48-years-old male).

*I did not tell other people that I am HIV positive. The way they [people in the community] speak, they mislead patients, they say bad things, that we sleep around with different men* (non-initiate, female, age unknown).

Among non-initiates, some were also concerned about the impact which disclosure may have had upon their family relationships.

*I think I need to have more time* *…* *If I can have more time I can be free to tell my parents like my mother… and my sisters and even my children. I just need to think on how I can guide my children on what the disease can do and how I can help them because the scary thing is that this disease kills* (non-initiate, 38-years-old male).

Fear of disclosure among non-initiates was compounded by difficulty in accepting their HIV-positive diagnosis. Both male and female respondents indicated that they needed time to come to terms with the new diagnosis. Most non-initiates indicated that they were still in shock and in disbelief regarding the diagnosis and were processing the news, limiting their ability to disclose and gain support from family and friends.

*As of now I cannot tell them [family]. I will tell them later…* *I am shocked as of now with the results that's why [I cannot disclose]* (non-initiate, 41-years-old female).

All initiates (both males and females) disclosed their HIV diagnosis to either sexual partners or immediate family members. Most female initiates disclosed their new status to extended family and friends with the hope that they would receive social support. Social support was usually provided in the form of emotional and psychological support and help navigating the health system. Support from extended family members and friends was almost exclusively mentioned by women; men rarely discussed social support from anyone except their sexual partner.

*I was disappointed [by the diagnosis] but I just accepted it because my relatives gave me hope when I told them* *…* *They can always take care of me in times of sickness and take me to the hospital and even give me the right medication since they know my sickness* (initiate, 21-years-old female)

#### Health system-level factors

None of the female respondents discussed a lack of privacy at the health facility as a barrier during the in-depth interview, while this issue was described as a major concern by some men. Men who reported lack of privacy as a barrier believed that accessing ART services automatically led to unwanted status disclosure because other clients or community members could see who accessed services at the ART clinic. This was especially true for facilities that only offered ART services on certain days or facilities without private ART waiting spaces.

*Since HIV testing is done privately and in confidence, receiving ARTs* *should also be done in a similar manner so that people can be able to take the drugs properly* *…* *All those on ARTs come either today or maybe tomorrow and receive their [ART] allocations one after the other. Privacy is compromised!* (non-initiate, 26-years-old male).

*Various people go to the hospital, including relatives* *…* *I asked them [providers],* *‘W* *here do you hold the ART lessons?* *’* *They just said,* *‘* *right here* *’* *…* *That got me worried because everyone is going to know that* *‘* *Oh, that group standing there is HIV positive* *’* (initiate, 26-years-old male).

The majority of men mentioned long waiting times and the need for frequent, repeat ART visits as barriers to ART initiation. This theme was not mentioned by women.

*There are not enough doctors to handle this issue [ART services]* *…* *They [providers] wronged us because they were taking too long* (non-initiate, 33-years-old male).

*Each time I come, the doctors are always busy… They told us to wait and the doctor would soon be with us. But sometime later we were told that we should go home because the doctor was still very busy* (non-initiate, 48-years-old male).

Time requirements to access ART services were further complicated by work demands experienced by men. Due to long waiting times, seeking ART services meant that men had to choose their health over income generation activities.

*What is making me fail [to initiate ART] is that I am busy* *…* *I become busy because I have cultivated some vegetables at the garden. I go in the morning and I get water from the well* (initiate, 38-years-old male).

## Discussion

In this paper, we use a mixed methods design to examine community- and health system-level factors associated with ART initiation among men and women in Malawi. We found that community and health system factors were associated with ART initiation for men and women in the era of universal treatment, even when controlling for individual and interpersonal factors.

At the community-level, receiving social support for accessing health services was positively associated with ART initiation for men but not for women. Qualitative findings show that both men and women valued social support to help them navigate the healthcare system, although only men mentioned support from their sexual partner and largely did not disclose their HIV status or rely upon friends and family for support with using HIV services. Men's limited disclosure of their HIV status to friends and family members has been reported throughout the region.^38^^–^^40^ Men traditionally occupy the role of breadwinner and rely on their spouse to facilitate family relations, which may limit men's ability to sustain meaningful social networks. Narrow gender norms of masculinity that require men to be strong and self-sufficient also contribute to men's fear of disclosure of their HIV status as this may reflect weakness and therefore tarnish their reputation among friends and members of the community.^41^

Fear of unwanted disclosure to a sexual partner was more commonly reported among ART non-initiates (compared with initiates) for both genders. Qualitative findings suggest that non-initiates were reluctant to disclose their HIV status because they feared stigma, an established barrier to treatment initiation.^17^^,^^42^ Other studies have reported high levels of HIV stigma, despite the recent expansion of ART.^43^ Continued efforts are needed to reduce HIV stigma. Programs that support HIV status disclosure and provide social support for service utilization may benefit both men and women. Men in particular may benefit from enhanced patient support services as they are less likely than women to have strong social networks outwith their sexual partner^44^ and are less likely to disclose their HIV status.^45^

At the health system-level, lack of privacy at the health facility was negatively associated with ART initiation among both men and women, while the belief that clients are turned away from health facilities without receiving care was also negatively associated with ART initiation for men. For both men and women, lack of privacy and unreliable service availability are widely cited as barriers to treatment throughout the region^46^ and must be addressed if universal treatment is to reach its full potential. Improved infrastructure may increase privacy of ART waiting spaces for both men and women, and integrated health services could reduce privacy concerns by making ART available alongside other non-HIV-related health services every day of the week. However, the implementation of integrated services remains a challenge in low-resource settings.^47^ Longer term strategies should focus on improving the efficiency and quality of HIV services, which would benefit both men and women. For men who have no other reason to visit the health facility, differentiated models of care such as community ART distribution and multi-month dispensing may help to address issues around privacy, long waiting times and unreliable services that interfere with their income generation activities.^22^

### Limitations

There are several limitations to note. First, our small sample size may have limited power to detect significant differences in the associations with gender and factors associated with ART initiation. Additionally, we only enrolled a small number of individuals who did not initiate ART and there were challenges in finding non-initiates to participate in the study, thus limiting the number of ‘cases’. This reduced the degrees of freedom for multivariable models and therefore limited the number of factors included in the multivariate model. Non-initiates who were successfully traced may be different to those who were not traced and may not be representative of all non-initiates. Second, we only had two female non-initiates in the qualitative sample. The limited sample size of female non-initiates precluded the ability to conduct a more robust analysis for this group and may compromise thematic saturation for this population. Study findings may also have limited generalizability to other clinics/facilities with different administrative and staffing infrastructure because our sample size is small, and we only included facilities with a high HIV burden. Finally, data may be sensitive to social desirability bias, particularly for health system factors, as respondents may wish to present a positive representation of their local health facility.

### Conclusion

We find that community and health system factors are associated with ART initiation in Malawi, even in the era of universal treatment, and that certain factors may be experienced or perceived differently by men and women. Interventions that reach across community and health system levels are required, and gender-tailored approaches should be explored further.

## Supplementary Material

ihaa041_Appendix
