# Supplementary material for: Community and health system factors associated with antiretroviral therapy initiation among men and women in Malawi: a mixed methods study exploring gender-specific barriers to care
Source: Int Health. 2020 Aug 25;13(3):253–61. doi: 10.1093/inthealth/ihaa041 (PMC8079311; doi:10.1093/inthealth/ihaa041)
Supplement: ihaa041_Appendix [file ihaa041_appendix.docx]

**Appendix A**

**Supplemental Table: Facility characteristics**

| **Facility** | **Facility Type** | **Facility Ownership** | **Location** | **Region** | **ART cohort size** |
| --- | --- | --- | --- | --- | --- |
| 1 | Mission Hospital | Private | Rural | Central | 1527 |
| 2 | Rural Hospital | Government | Peri – Urban | Central | 1783 |
| 3 | Health Centre | Government | Rural | Central | 227 |
| 4 | Health Centre | Government | Rural | Central | 747 |
| 6 | Health Centre | Private | Rural | Central | 1119 |
| 7 | Health Centre | Government | Rural | South | 736 |
| 8 | Health Centre | Government | Rural | South | 644 |
| 9 | Health Centre | Government | Rural | South | 553 |
